# Insights into the pattern of choroidal vascularity index changes in idiopathic macular hole

**DOI:** 10.1038/s41598-024-51739-8

**Published:** 2024-01-11

**Authors:** Huaqin Xia, Jiarui Yang, Qingyi Hou, Xinchun Wu, Changguan Wang, Xuemin Li

**Affiliations:** 1 Department of Ophthalmology, Peking University Third Hospital, Beijing, China; 2 Beijing Key Laboratory of Restoration of Damaged Ocular Nerve, Peking University Third Hospital, Beijing, China

**Keywords:** Diseases, Medical research

## Abstract

This retrospective study aimed to investigate the changes in choroidal vascularity index (CVI) before and after surgery for idiopathic macular hole (MH). Enhanced depth imaging optical coherence tomography (EDI-OCT) images were analyzed at baseline and at 1-week, 1-month, and 3-month postoperative visits. A total of 97 patients (97 eyes) were included in the study. At baseline, overall CVI and macular CVI showed negative correlation with axial length (AL) and positive correlation with central corneal thickness (CCT). There were no significant differences in macular CVI or overall CVI between affected and healthy eyes, as well as in subgroup analysis of different stages of macular CVI. Following surgery, there was a significant decrease in CVI at 1 week postoperatively, followed by a gradual recovery to baseline levels over time. The observed changes in CVI may be attributed to factors such as air tamponade, pressure changes, and photoreceptor metabolism. This study provides insights into the pattern of CVI changes associated with MH surgery. The findings suggest that stage 4 MH is associated with decreased macular CVI in affected eyes. These results contribute to a better understanding of the effects of surgery on choroidal blood flow in MH patients.

## Introduction

Idiopathic macular hole (MH) is a vitreomacular disease characterized by a complete absence of retinal tissue in the macula, posing a significant threat to visual acuity. Although the exact pathogenesis of idiopathic MH remains unclear, it is widely accepted that it is associated with tangential traction exerted by epiretinal membrane (ERM) or the vitreous in front of the macula^1^. At present, structural parameters such as minimum linear distance (MLD) and disruption of the ellipsoid zone (EZ) are utilized to evaluate the status of MH and predict its prognosis^2^. However, there is a lack of research on blood flow-related parameters.

The Choroidal Vascularity Index (CVI), a novel parameter proposed by Branchini et al.^3^, is used to evaluate the status of choroidal blood flow. On optical coherence tomography (OCT) images, the vascular region of the choroid is visualized as black pixels. By binarizing the images, the ratio of black pixels to the total number of pixels in the choroidal region can be calculated as a percentage, providing information about the choroidal vascular region and indicating the status of choroidal blood flow. Currently, CVI has been extensively studied in conditions such as diabetic retinopathy (DR)^4–6^, age-related macular degeneration (AMD)^7,8^ and central serous chorioretinopathy^9,10^. Furthermore, it is anticipated that CVI will have further clinical applications in the treatment of retinal and choroidal diseases.

Given the widespread use of CVI in other vitreoretinal diseases and the lack of blood flow-related parameters in MH, we believe it is beneficial to explore the pattern of changes in CVI during MH treatment. Thus, our aim was to examine the pattern of CVI changes before and after MH surgery, serving as an indicator of alterations in choroidal blood flow.

## Results

### Basic characteristics

Nighty-seven eyes (51 OD, 46 OS) of 97patients (26 male, 71 female) were included in this study (Table 1). The mean age was 61.15 ± 11.21 years, and the proportion of stage 2 MH was 11.34%, stage 3 was 20.62% and stage 4 was 68.04%. The mean reported duration of MH was 141.7 ± 163.9 days (7–365) in all patients except one patient with a duration of 7 years and two patients with a duration of 3 years. The patients had a MLD of 407.36 ± 207.58 um, a preoperative macular CVI of 64.88 ± 2.97, and overall CVI of 64.60 ± 2.63. 61 of the 97 patients underwent cataract surgery simultaneously. The patients’ mean preoperative IOP was 14.78 ± 2.95 mmHg.

**Table 1:** Baseline parameters of MH patients.

Item	Value
Number of eyes	97
Sex, M/F	26/71
Eye, OD/OS	51/46
Axial length, mm	23.90 ± 1.52
Age, years	61.15 ± 11.21
MH stages, n (%)	
2	11 (11.34%)
3	20 (20.62%)
4	66 (68.04%)
BCVA, LogMAR	1.01 ± 0.40
MLD, µm	407.36 ± 207.58
BD, µm	826.70 ± 376.71
H, µm	373.52 ± 77.50
ELM disruption, µm	879.37 ± 402.16
EZ disruption, µm	943.45 ± 305.92
Macular CVI	64.88 ± 2.97
Mean CVI	64.60 ± 2.63
CCT, µm	181.48 ± 66.47
Duration, days	196.02 ± 387.874
Phaco, Y/N	61/36
IOP, mmHg	14.78 ± 2.95

All values are the mean ± standard deviation unless otherwise indicated.

MH, macular hole; BCVA, best corrected visual acuity; MLD, minimal linear diameter; BD, basal diameter; H, height; EZ, ellipsoid zone; ELM, external limiting membrane; CVI, Choroidal vascularity index; CCT, central choroid thickness; Phaco, phacoemulsification; IOP, intraocular pressure.

### Correlation of baseline parameter with CVI

We explored the correlation between CVI and preoperative parameters, and bivariate correlation analysis revealed that overall CVI and macular CVI were not significantly correlated with VA (*P* = 0.114; *P* = 0.261), IOP (*P* = 0.394; *P* = 0.764), mean MLD (*P* = 0.773; *P* = 0.577), and mean diameter of EZ disruption (*P* = 0.472; *P* = 0.889). While both overall CVI and macular CVI were negatively correlated with the AL (*P* < 0.001; *P* = 0.017) and positively correlated with CCT (*P* < 0.001; *P* < 0.001) (Table 2).

**Table 2:** Correlation of baseline parameter with CVI.

		Overall CVI	Macular CVI
Visual acuity	CC	0.17	0.12
	*P*	0.114	0.261
IOP	CC	− 0.09	− 0.03
	*P*	0.394	0.764
Axial length	CC	− 0.44	− 0.28
	*P*	< 0.001***	0.017*
Mean MLD	CC	0.03	− 0.06
	*P*	0.773	0.577
Mean EZ disruption	CC	0.07	− 0.01
	*P*	0.472	0.889
CCT	CC	0.523	0.506
	*P*	< 0.001***	< 0.001***

All values are the mean ± standard deviation unless otherwise indicated.

IOP, intraocular pressure; MLD, minimal linear diameter; EZ, ellipsoid zone; CCT, central choroid thickness; CC, correlation coefficiency.

P < 0.05 is marked with *, P < 0.01 is marked with ** and P < 0.001 is marked with ***.

### CVI comparison of different stages of MH

Further we analyzed whether there were differences in baseline CVI of different stage of MH and with contralateral healthy eyes. The results showed no difference of macular CVI (*P* = 0.094) and overall CVI (*P* = 0.974) between affected eyes and healthy eyes. Subgroup analysis were performed afterwards, the results showed that macular CVI of stage 2 MH was 64.38 ± 3.57, stage 3 was 65.67 ± 2.46, and stage 4 was 64.65 ± 3.08, with no statistically significant difference between each two of them (2 vs 3: *P* = 0.166; 3 vs 4: *P* = 0.193; 2 vs 4: *P* = 0.553). Overall CVI of stage 2 MH was 64.08 ± 2.98, stage 3 was 65.40 ± 2.24, and stage 4 was 64.42 ± 2.71, with no statistically significant difference between each two (2 vs 3: *P* = 0.143; 3 vs 4: *P* = 0.270; 2 vs 4: *P* = 0.409). For contralateral healthy eyes, macular CVI of stage 2 (*P* = 0.767), stage 3 (*P* = 0.847) and stage 4 (*P* = 0.040) showed no significant difference. Overall CVI of stage 2 (*P* = 0.692), stage 3 (*P* = 0.487) and stage 4 (*P* = 0.514) showed no significant difference (Table 3).

**Table 3:** Preoperative CVI comparison of different stages of MH.

		Macular CVI	*P* value	Overall CVI	*P* value
Baseline overall	Healthy eye	65.40 ± 3.13	0.094	64.59 ± 2.83	0.974
	Affected eye	64.83 ± 3.02		64.58 ± 2.66	
Stage 2 (N = 11)	Healthy eye	64.15 ± 3.07	0.767	63.72 ± 3.32	0.692
	Affected eye	64.38 ± 3.57		64.08 ± 2.98	
Stage 3 (N = 20)	Healthy eye	65.83 ± 2.50	0.847	64.97 ± 2.07	0.487
	Affected eye	65.67 ± 2.46		65.40 ± 2.24	
Stage 4 (N = 66)	Healthy eye	65.50 ± 3.30	0.040	64.64 ± 2.95	0.514
	Affected eye	64.65 ± 3.08		64.42 ± 2.71	

All values are the mean ± standard deviation unless otherwise indicated.

CVI, Choroidal vascularity index; P < 0.025 was considered statistically significance for Bonferroni correction was applied.

### Change of CVI after the surgery

**Figure 1:**
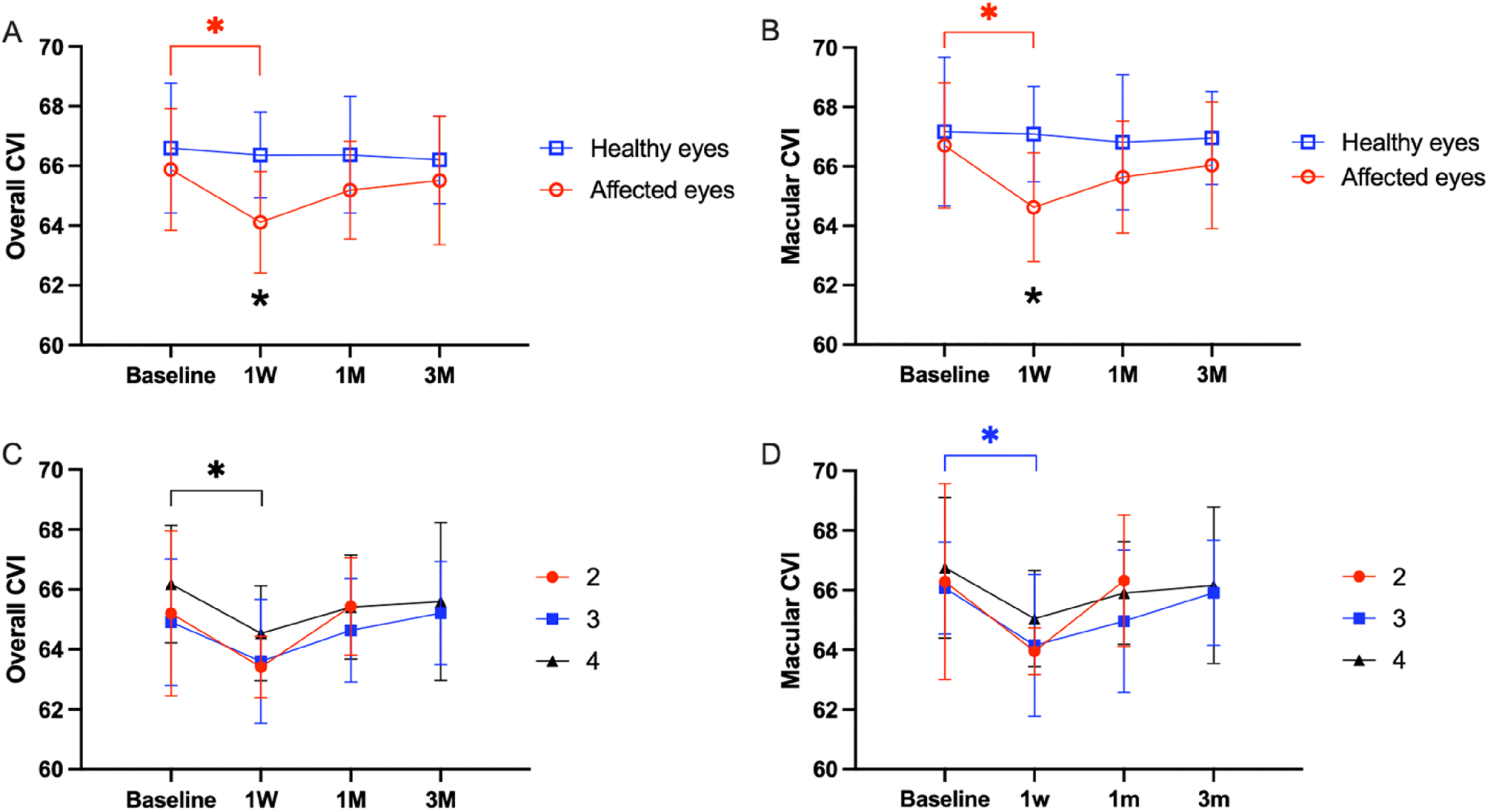
Change of CVI after the surgery. (**A**) Overall CVI change after the surgery in affected and healthy eyes; (**B**) Macular CVI change after the surgery in affected and healthy eyes; (**C**) Overall CVI change after the surgery indifferent stages of MH; (**D**) Macular CVI change after the surgery in indifferent stages of MH.

To investigate the change of CVI after the surgery, we performed a one-way repeated-measures ANOVA with the AL defined as a covariate to eliminate the effect of AL on the results. For repeated measures ANOVA requires complete data from the four follow-up visits at baseline, 1 week after, 1 month after, and 3 months after surgery, a total of 54 individuals were included in this analysis after excluding incomplete data. The results showed that there was a difference in overall CVI at the four time points after excluding the effect of AL in the affected eyes (P = 0.043), and the post hoc test revealed a significant decrease in macular CVI at 1 week postoperatively compared to preoperatively (P = 0.034). The Macular CVI analysis showed that macular CVI at the four time points differed after excluding the effect of the AL in the affected eyes (P = 0.005), and the post hoc test showed that the macular CVI decreased significantly at 1 week postoperatively compared to the preoperative period (P = 0.019). In contrast, there was no significant difference in overall CVI and macular CVI in healthy eyes at the four time points after removal of AL influence (P = 0.373, P = 0.547). We further explored CVI changes in different stages of MH. The results showed that the changes in CVI of grade 2, 3, and 4 MH all showed a decreasing trend at 1 week postoperative. Compared with the baseline, although only macular CVI of stage 3 MH (*P* = 0.041) and overall CVI of stage 4 MH (*P* = 0.011) showed a statistically significant decrease at 1 week, the macular CVI of stage 4 MH was close to the edge of a statistically significant difference (*P* = 0.065) (Fig. 1).

## Discussion

The introduction of enhanced depth imaging optical coherence tomography (EDI-OCT) has provided clearer choroidal images and enabled the analysis of choroidal blood flow. Extensive research has been conducted on CVI in various diseases such as AMD^7,8^, DR^4–6^ and uveitis^11–13^. Our previous studies have demonstrated that CVI shows promise as a diagnostic and monitoring parameter for myopic choroidal neovascularization^14^, and as a predictor of visual prognosis following ERM surgery^15^. However, the impact of MH surgery on CVI remains inconclusive, necessitating the present study. Our findings indicate that baseline CVI does not significantly differ across different stages of MH, but macular CVI of stage 4 MH was larger in affected eyes compared with contralateral healthy eyes. Moreover, CVI experiences a significant decrease in the early postoperative period (1 week) after surgery, followed by gradual recovery to baseline levels.

In terms of preoperative CVI, our results revealed no significant difference between affected and healthy eyes, nor was there a significant difference in CVI between different stages of MH. However, subgroup analysis showed that *P* value of macular CVI between affected and healthy eyes in stage 4 MH was close to cut-off value (0.04 vs 0.025). Limited studies have investigated preoperative CVI in MH. Rizzo et al.^16^ analyzed seven MH patients and reported a significantly higher CVI in the affected eye compared to the contralateral healthy eye (*P* = 0.004). These researchers proposed that vitreomacular traction affects choroidal vascular structure through various mechanisms, resulting in differences in CVI between affected and healthy eyes. Conversely, Chun et al.^17^ compared the CVI of the affected eye with contralateral healthy eye in 51 patients with unilateral MH and found no significant difference between the two (*P* = 0.253). Similarly, Ercan et al.^18^ enrolled 56 patients with unilateral MH and found no significant difference in CVI between the affected eye and the contralateral healthy eye (*P* = 0.81). Our results demonstrated that stage 4 MH got a trend of decrease in macular CVI. As photoreceptors are highly metabolically active^19^, and 90% of the oxygen they consumed came from choroid circulation^20^. We speculated that the mechanism for the possible decrease in CVI in the macular region of stage 4 MH lies in the defect of the entire retinal layer in the macular region, resulting in a decrease in metabolic demand. Therefore, a consequent decrease in the choroidal blood flow that feeds blood to the region may have occurred.

Regarding the postoperative recovery process, we observed a decrease in CVI followed by recovery in eyes with MH, with the lowest CVI recorded at 1 week postoperatively. However, we did not observe any significant change in CVI in the contralateral healthy eye. This trend aligns with previous studies. Rizzo et al.^16^ demonstrated a gradual reduction in CVI after vitreomacular disease, with CVI values of 63.85 ± 4.04, 62.45 ± 2.25, and 61.06 ± 3.79 at baseline, 1 month postoperatively, and 3 months postoperatively, respectively. The lowest point was observed at 3 months postoperatively. The results of Ercan et al.^18^ also found lower CVI values at 8 weeks postoperatively in both MH- and ERM-affected eyes compared to preoperative values (*P* < 0.001; *P* < 0.001). Our findings are consistent with these previous studies, which indicated a decreasing trend in CVI following surgery. However, our study introduced a new time point for assessment, specifically 1 week postoperatively, and identified a more significant decrease in CVI during the early postoperative period. This change was not reflected in earlier studies.

We hypothesized that the most likely cause of the decreased CVI at 1 week postoperatively was physical stress. Previous research has tried to explore the change of ocular blood flow following vitrectomy, which may help to explain our result. Okamoto et al.^21^ using laser speckle flowgraphy, observed a significant decrease in intraoperative blood flow, possibly due to intraoperative infusion pressure. This result demonstrated that physical pressure has a significant effect on ocular blood flow. In our study, MH patients underwent air tamponade and maintained a prone position for at least 5 days. During the first postoperative week, air filling the vitreous body presses against the retina, promoting hole closure and exerting corresponding pressure on the choroid, resulting in decreased choroidal blood flow, as reflected by reduced CVI. As for the rebound of CVI at 1 month postoperatively, we believe that it can be explained by both extrinsic and intrinsic factors. In terms of extrinsic factors, after the first week, as sterile air gradually absorbed, the physical pressure on the retina and choroid disappears, leading to the rebound of CVI. And in terms of intrinsic factors, we hypothesis that need for oxygen supply played an important role. Siegfried et al.^22^ found increased pO_2_ levels in the posterior chamber of patients following vitrectomy, suggesting that alterations in post-vitrectomy oxygenation could also impact choroidal blood flow. Previous study revealed that photoreceptors consumed 90% of the oxygen delivered to the retina to maintain ion homeostasis, and 90% of the oxygen was delivered by choroidal circulation^19^. And maintaining the steep gradient of oxygen tension requires high blood flow in the choroid^23^. Therefore, the restoration of the structure causes an increase in the demand for oxygen, and thus regulating the blood supply in accordance with the demand for oxygen, which further leads to an increase in the blood flow supply. However, all of the above conjectures are based on our clinical experience, and thus more in-depth studies are needed in the future to clarify the mechanism of change in CVI.

Several limitations should be acknowledged in this study. Firstly, this was a retrospective single-center study with a relatively small sample size, thus the results of the subgroup analysis were affected by the small amount of data from the three-month postoperative follow-up of stage 2 MH patients. Future studies should include more data from multiple centers and operators to strengthen the findings. Secondly, our study only included patients who underwent classical ILM peeling, lacking those who underwent modified procedures such as ELM reversal tamponade.

In conclusion, our study found stage 4 MH exhibit lower macular CVI compared with healthy eyes. Postoperatively, CVI exhibited a decreasing trend followed by gradual recovery in the affected eyes, indicating vitrectomy indeed affect choroidal blood flow in MH patient. And future studies are needed to explore the predictive value of CVI in MH patients.

## Method

### Patient

It was a retrospective study approved by the Institutional Review Board of Peking University Third Hospital and conducted in accordance with the tenets of the Declaration of Helsinki. Informed consent was obtained from all subjects. Subjects meeting specific criteria were enrolled: they had been diagnosed with idiopathic macular hole between 2018 and 2021, underwent surgery at our center and acquired successful hole closure afterwards (MH closed at 1w, 1 m and 3 m postoperative visit), and had preoperative and postoperative enhanced depth imaging optical coherence tomography (EDI-OCT) scans. Individuals with other retinal diseases such as diabetic retinopathy or retinal vein occlusion, as well as those who had previously undergone retinal surgery, were excluded from the study. Axial length measurements were taken using the IOL Master Biometry 700 (Carl Zeiss Meditec, Jena, Germany). Best corrected visual acuity (BCVA) was assessed at each visit and converted to logMAR units for statistical analysis. Intraocular pressure (IOP) was measured using the Topcon CT-800 non-contact tonometry (Topcon, Tokyo, Japan) at baseline and during each postoperative visit. Demographic information was obtained from medical records.

All patients underwent 25-gauge pars plana vitrectomy performed by a senior surgeon. Simultaneous cataract phacoemulsification and intraocular lens implantation were performed if there was lens opacity. Indocyanine green (0.25%) diluted in 10% glucose was utilized to assist in internal limiting membrane peeling, with a peeling radius of 2 disc diameters. Complete fluid-air exchange was conducted afterwards with tamponade of sterile air, and no expanding gas was used. Patients were instructed to maintained a prone position for a minimum of 5 days.

### OCT data acquisition and processing

Baseline, 1-week, 1-month, and 3-month postoperative EDI-OCT scans were performed using the Spectralis OCT(Heidelberg Engineering, Heidelberg, Germany). Only images with a strength index greater than 25 were included in the analysis. At each visit, two B-scan images passing through the fovea were acquired with a 9 mm scan in both horizontal and vertical directions.

Central choroid thickness (CCT) was manually measured by averaging measurements taken along the horizontal and vertical axes from the inner to the outer edge of the choroid at the foveal center. Furthermore, various parameters such as minimum linear diameter (MLD), basal diameter (BD), height (H) of MH, diameter of ELM disruption, diameter of EZ disruption, and EZ-related angle were manually measured using ImageJ software (version 1.47v, Wayne Rasband, National Institutes of Health, Bethesda, MD, USA, http://imagej.nih.gov/ij). For MLD, BD, H of MH, diameter of ELM disruption, and diameter of EZ disruption, measurements were obtained on both the horizontal and vertical axes, and their averages were calculated. Two staff members performed all measurements, and the final values were averaged.

### Measurement of CVI

**Figure 2:**
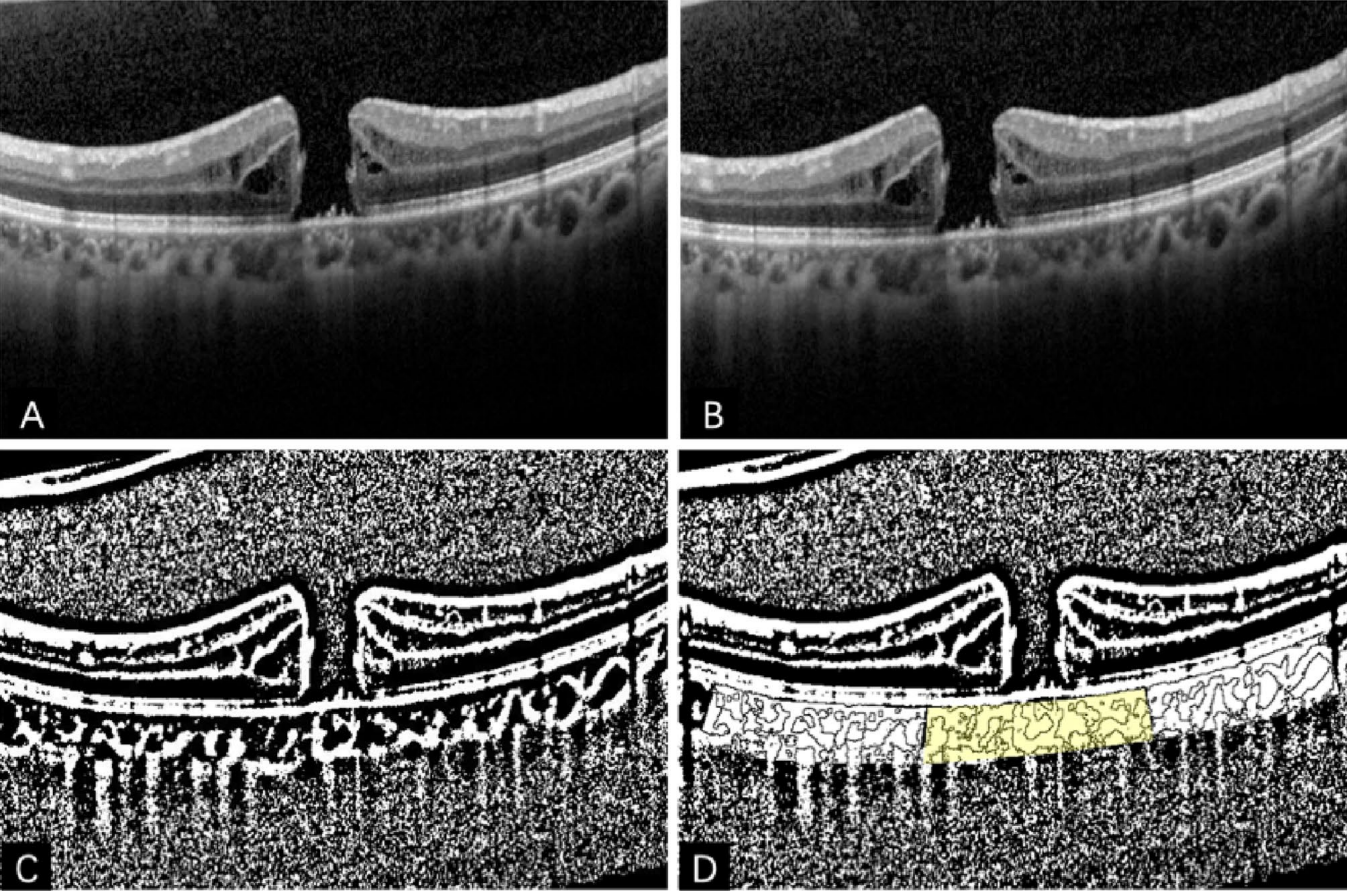
Binarization of OCT image. (**A**) the original image of the RGB color format; (**B**) image converted to 8-bit format; (**C**) binarization by applying Niblack auto local threshold; (**D**) segmentation of choroid, yellow marked macular CVI and white marked temporal, nasal, superior, and inferior sides according to the direction of image, with 2 mm length for each region.

CVI was manually measured using ImageJ software^24^. The images were imported into the software and converted to 8-bit grayscale. Niblack auto local threshold was applied to binarize the images (Fig. 2). After binarization, black pixels represented the vascular zone of the choroid. Hence, the CVI value was defined as the ratio of black pixels to the area occupied by the choroid. Manual labeling was used to identify the choroidal area, and the CVI of the choroid was measured within a range of 2 mm below the fovea and 1–3 mm on either side of the fovea. Measurements of CVI were taken in the macular, temporal, nasal, superior, and inferior sections based on the orientation of the B-scan images. The average of the vertical and horizontal measurements of the fovea represented the macular CVI value. The overall CVI value was calculated as the average of the six measurements from vertical and horizontal orientation, including macular, temporal and nasal region from horizontal orientation and macular, superior and inferior region from vertical orientation. Two staff members performed all measurements, and the final values were averaged.

### Statistical analysis

IBM SPSS for Mac version 26.0 (IBM Corp., Armonk, NY, USA) was utilized for statistical analyses. Continuous values were presented as mean ± standard deviation (SD). Bivariate correlation analysis was employed to assess the correlation between baseline CVI and baseline parameters. Paired t-test was used to explore the difference in baseline CVI between healthy eye and affected eye with Bonferroni correction if needed. Analysis of covariance was used to explore the differences in CVI at various stages of MH. Repeated analysis of variance was applied to investigate the trends of CVI changes at baseline and during postoperative visits. The significance level was set at *P* < 0.05 unless stated otherwise.

## Data Availability

The data that support the findings of this study are available from the corresponding author Xuemin Li, upon reasonable request.
